# Testing Claims of Crime-Related Amnesia

**DOI:** 10.3389/fpsyt.2018.00617

**Published:** 2018-11-21

**Authors:** Marko Jelicic

**Affiliations:** ^1^Forensic Psychology Section, Department of Clinical Psychological Science, Faculty of Psychology and Neuroscience, Maastricht University, Maastricht, Netherlands; ^2^Department of Criminal Law and Criminology, Faculty of Law, Maastricht University, Maastricht, Netherlands

**Keywords:** crime-related amnesia, deception, feigning, malingering, forensic neuropsychology

## Abstract

Many violent offenders report amnesia for their crime. Although this type of memory loss is possible, there are reasons to assume that many claims of crime-related amnesia are feigned. This article describes ways to evaluate the genuineness of crime-related amnesia. A recent case is described in which several of these strategies yielded evidence for feigned crime-related amnesia.

## Introduction

A few years ago, 29 year old Randy unexpectedly appeared at the house of his parents. Because he was covered in blood, his father asked him if something happened to his girlfriend. Randy nodded, upon which his father called the emergency number. The police speeded to Randy's apartment and found his girlfriend lying on the floor in a pool of blood. She had been stabbed to death. Randy was arrested and taken to the police station. During his interrogation, he told the police that, although he did not rule out having killed his girlfriend, he had no memory for this fatal incident whatsoever.

Randy's case is not unique: a nontrivial percentage of people who are accused or convicted of violent offenses claim crime-related amnesia. About 70 years ago, Leitch (1) found that 16 out of 51 offenders (31%) convicted of homicide reported memory loss for their crime. Several decades later, Taylor and Kopelman (2) interviewed 203 men charged with both violent and non-violent offenses. Of the 34 men accused of having committed murder or manslaughter, 9 of them (26%) claimed amnesia for their crime. More recently, Pyszora et al. (3) studied the case note-notes of 207 individuals sentenced to life imprisonment. In this sample, 60 (29%) reported memory loss for their offense. By and large, it seems that about 20 to 30% of those who have committed violent crimes claim crime-related amnesia (4). It should be noted here that this form of memory loss is not only reported by violent offenders: individuals convicted for sexual and property offenses also claim amnesia for their crimes (5).

Apparently, a considerable number of people—both laypersons and professionals—believe that offenders can forget or repress a serious crime that they committed. Magnussen et al. (6) asked 1,000 Norwegians whether or not murderers who claim amnesia for their offense are telling the truth about their memory loss. Thirty nine percent of the respondents opined that such offenders are truthful about their amnesic episode. In a follow-up study, Magnussen and Melinder (7) asked 857 Norwegian licensed psychologists, most of them working in the field of clinical psychology, for their opinion about this issue. Thirty eight percent of this sample of professionals endorsed the view that murderers who claim crime-related amnesia are honest about the gap in their memory. More recently, Melinder and Magnussen (8) asked 117 psychiatrists and psychologists who served as expert witnesses in Norwegian courts whether or not murderers who report crime-related amnesia are telling the truth about their memory loss. This time, 39 percent of the respondents indicated that such offenders are truthful about their amnesia. Because these studies were all conducted in Norway, one could argue that these findings may not be generalized to countries outside Scandinavia. However, according to Lynn et al. (9), the belief that offenders can repress crime-related memories appears to be a worldwide phenomenon.

## What does science say about crime-related amnesia?

There are three different explanations for memory loss in offenders. The first explanation contends that, during the time of the crime, some offenders suffer from a temporary (or permanent) brain dysfunction that prevents or undermines the storage of criminal events in memory. This type of memory loss is labeled organic amnesia (4). The second explanation holds that many offenders are in an extreme emotional condition (e.g., rage) when committing a violent crime. Therefore, crime-related details would be stored in memory in the context of strong emotions. Later, when the offender has returned to a more calm state of mind, he or she would be unable to remember the crime because of a mismatch in emotional state between the encoding of crime-related events and the retrieval of such events. This type of memory loss is termed dissociative amnesia (4). When people have (dissociative) amnesia for a crime of passion, some authors prefer to speak of a “red-out” (10). The third explanation for crime-related amnesia is that a considerable number of offenders are pretending to be unable to remember crime-related details. This type of memory loss is called feigned amnesia (4).

Temporary brain dysfunction can lead to crime-related amnesia. The thalamus, hippocampus, and prefrontal cortex are all involved in the encoding and storage of information in autobiographical memory (11). Out of these three brain areas, the hippocampus is probably the most vulnerable to temporary or permanent dysfunction. Closed head injury, consumption of large quantities of alcohol, use of certain prescription or illegal drugs, low blood sugar (hypoglycemia), as well as shortage of oxygen (hypoxia) may result in a temporary deranged hippocampus (12). A considerable portion of offenders who claim crime-related amnesia report that their inability to recollect criminal events is due to alcohol consumption (5, 13). However, drinking alcohol does not necessarily lead to amnesia. In order to develop an alcohol blackout, one should drink large quantities of alcohol. This type of memory loss is assumed to be only plausible when the blood alcohol concentration (BAC) of the offender is higher than 0.25% (14). While most medications do not affect memory, prescription drugs that do have amnesic side effects include benzodiazepines and other hypnotics, antidepressants, and anticonvulsants (15). Gamma Hydroxybutyrate (GHB) is an illegal drug that can lead to temporary memory loss (16).

There are several reasons to doubt the existence of dissociative amnesia for an offense. For one thing, laboratory studies in which participants encode and store information in a particular emotional state and retrieve that information in another state have shown that a mismatch in state between the acquisition and test phase does not lead to a substantial inability to remember stimuli presented in the learning phase (17). Also, committing a (violent) crime typically means that one performs one or more actions. Research has shown that people tend to remember their own actions better than other information (18). Most importantly, dozens of studies have demonstrated that strong emotions do not undermine memory performance, but enhance memory for stressful events (19, 20). A recent Canadian study serves as a case in point. McKinnon et al. (21) investigated the richness and accuracy of people's memory for a highly traumatic event. Their participants were former passengers of a transatlantic plane flight that nearly ditched at sea. The authors found that, a few years after the incident, all participants had excellent memory for events that took place during the near-fatal flight. Based on this investigation and many other studies showing memory enhancement by strong emotions, one could reason that dissociative amnesia for an offense is, at best, scarce. This notion has also been put forward by some forensic psychologists. Centor (22), for example, stated: “My own experience, during a period of over 11 years in a forensic unit, failed to confirm even one case of psychogenic amnesia in the absence of a psychotic episode, brain damage, or acute brain syndrome” (p.240).

Crime-related amnesia clearly has benefits for people charged with serious offenses (23). To start with, one cannot provide the police with crucial details of an offense, which might obstruct police investigations. Also, sexual offenders do not have to talk about a shameful offense. In addition, having no memory for a crime suggests that the offense was impulsive and not premeditated (in homicide cases, this could lead a manslaughter instead of a murder conviction). Moreover, this type of amnesia might lead to a mitigation of criminal responsibility. Given these advantages, it seems likely that many offenders who report memory loss for their offense are actually feigning their amnesia. A famous historical example of feigned crime-related amnesia is that of Rudolf Hess. This prominent Nazi politician claimed, at the start of the Nuremberg trials, to have no recollections of his personal and political activities in the years preceding the Second World War. Hess was examined by a number of psychiatrists who unanimously declared that his memory loss was genuine. However, when after some weeks, Hess realized that, because of his amnesia, he could not respond to the allegations against him, he informed the tribunal that he had feigned his memory loss (24).

## Evaluating the veracity of crime-related amnesia

As mentioned above, a dysfunctional hippocampus can lead to impaired memory storage. Therefore, when asked to evaluate the authenticity of a claim of crime-related amnesia, the first thing a forensic psychologist or psychiatrist should do is to determine if organic factors might account for the putative memory loss reported by the offender (25). To establish whether or not the offender had a deranged hippocampus because of excess consumption of alcohol, it would be wise to calculate his or her BAC level (26). This is not a hard thing to do: many BAC calculators can be found on the Internet. Given the questionable status of dissociative amnesia, crime-related amnesia reported by an offender without hippocampal dysfunction at the time of the crime should be treated with skepticism (22).

Clinical features of the memory loss reported by the offender may shed light on the genuineness of amnesia. Power (27) argued that periods of real memory loss have a gradual and blurred onset and termination. Thus, an amnesic episode with an abrupt beginning and end would be suggestive of feigned memory loss. Moreover, people with true amnesia usually have “islands of memory” (28). That is, they do not have complete memory loss, but are still able to remember elements of events that occurred during their amnesic period. Hence, absolute amnesia would be indicative of feigned memory loss, while a “patchy” amnesia suggests bona fide memory loss. Note that in people with mild head injury or alcohol intoxication, there usually is shrinkage of their amnesia (29). At first, such individuals cannot remember events that took place in the days (or sometimes weeks) before the injury or intoxication. However, as time passes by, their memories of these events gradually return. Typically, old memories return before more recent recollections, a phenomenon called “Ribot's law”—named after the nineteenth century French psychologist Théodule Ribot (30). Thus, shrinkage of amnesia is line with a genuine inability to remember certain criminal events. Schacter (31) stated that feelings-of-knowing rating might also be used as an indicator of the veracity of crime-related amnesia. Feelings-of-knowing pertain to the idea that, when unable to remember autobiographical events, one could retrieve information from memory when given the right hints or cues. Because true amnesia often goes hand in hand with a feeling-of-knowing, an offender stating that not even hypnosis or truth serum will bring back crime-related events, would be suggestive of feigned memory loss. Although Schacter's suggestion is interesting, some authors are critical about the use of feelings-of-knowing as a tool to determine the authenticity of crime-related amnesia (32). In a number of cases, clinical features of the alleged memory loss may not provide the forensic psychologist or psychiatrist with valid information pertaining to the credibility of crime-related amnesia. Research suggests that a large percentage of offenders have a history of traumatic brain injury (33). Because such offenders have intimate knowledge of genuine temporary memory loss, forensic psychologists and psychiatrists should be cautious to use clinical features of crime-related amnesia as evidence for true memory loss.

Using standard questionnaires and tests designed to measure a tendency to feign memory problems is another strategy to determine the authenticity of crime-related amnesia. An example of such a questionnaire is the Structured Inventory of Malingered Symptomatology (34). The SIMS is a self-report instrument determining feigning of psychiatric symptoms and cognitive impairments. It comprises 75 yes/no items that measure an individual's proneness to endorse bizarre and/or atypical symptoms in five different areas including amnesia. The rationale behind the instrument is that feigners do not know how genuine symptoms manifest themselves. Examples of items from the amnesia subscale are “Recently I've noticed that my memory is getting so bad that there have been entire days I cannot recall” and “At times I've been unable to remember the names and faces of close relatives so that they seem complete strangers.” Each improbable item that is endorsed is scored “1.” Scores on the 75 items are added up to obtain a total SIMS score. A score of 17 or higher is considered indicative of feigning of symptoms (35)—although some authors have argued that a higher cutting score should be used (36). The SIMS has acceptable psychometric properties (37). As mentioned above, the SIMS consists of items pertaining to improbable symptoms. A potential limitation of questionnaires that only list bizarre and/or improbable symptoms is that they might be easily identifiable as tests measuring feigning. For that reason, Merten et al. (38) developed the Self-Report Symptom Inventory (SRSI) to determine feigning of different psychiatric disorders and/or cognitive impairment. In contrast with the SIMS, the SRSI consists of items that ask for pseudo-symptoms and genuine symptoms. Although the SRSI seems to have promising psychometric characteristics, more research on the diagnostic accuracy of this instrument is necessary before it can be used in forensic practice.

A well-known example of a test developed to measure feigned memory impairments is the Test of Memory Malingering (TOMM). This test may also be used to investigate the veracity of crime-related amnesia (39). The TOMM is an easy memory test requiring only passive recognition. The idea behind this test is that genuine brain-disordered patients perform quite well on it. Because feigners want to convince the forensic psychologist or psychiatrist that they suffer from memory problems, they often perform substantially poorer on the TOMM than bona fide patients with memory disorders. The TOMM contains two learning trials where the examinee is shown 50 line drawings of common objects. Both trials are followed by a forced choice recognition task. A retention trial given 15 min after the second learning trial consists of the forced choice recognition task only. For each correct answer, the item is scored “1.” A score below 45 on the second learning trial or the retention trial is considered indicative of feigned memory impairments. A number of studies have shown that the TOMM has good psychometric properties (40, 41). Besides the TOMM, there are other well-validated tests that can be used to evaluate an individual's tendency to feign memory problems, such as the Amsterdam Short-Term Memory Test (42) and the Word Memory Test (43).

A drawback of the above-described questionnaires and tests is that they can only be used in cases where the offender claims that his or her inability to remember crime-related details is the result of a general memory deficit due to, for instance, sleeping problems, use of certain prescription drugs or a neurological disorder. These instruments do not work in offenders who say that normally they have no memory problems, but because of excessive drinking and/or taking illegal drugs on the day of the offense they cannot remember criminal acts. In such cases, symptom validity testing might be helpful in assessing the authenticity of claims of crime-related amnesia.

Symptom validity testing (SVT) was originally created to assess the credibility of hearing problems (44). More recently, it has been used as an instrument to assess the veracity of crime-related amnesia (45, 46). SVT consists of a forced choice technique in which an offender who claims to suffer from crime-related amnesia is asked a range of questions pertaining to details of the crime and/or crime scene (47). For each question, the examinee must choose between two equally plausible answers, one of which is correct and the other is incorrect. True memory loss for a crime should result in random performance on the SVT. Or in other words, bona fide amnesia will result in ~50% of the answers being correctly answered. If significantly more incorrect answers are given than correct answers, an offender is performing below chance level performance. This can only be achieved when one is intentionally giving incorrect answers, which is indicative of having preserved memory for criminal events. Because SVT is based on binomial statistics, the exact probability of a deviant memory performance can be quantified (see case below). Unfortunately, SVT can only be used in a limited number of cases. One needs to be able to create a substantial number of two choice questions about the crime and/or crime scene from the investigative reports. In addition, in a proper SVT procedure, only the offender and the police should have intimate knowledge of the crime. If details of the crime have been “leaked” to the offender via the media, police officers or his or her attorney, the offender might claim amnesia and at the same time legitimize an above-chance level on the SVT by referring to the media, police officers or his or her attorney.

It should be noted here that offenders who feign amnesia for a crime are lying about their memory loss. For that reason, psychophysiological and neural measures created to detect lying (48) might also be used to evaluate the authenticity of a crime-related amnesia claim. However, these measures have not yet been used in forensic practice.

## Case

This article started with the case of Randy who claimed to have no memory of the stabbing of his girlfriend. At the time of the offense, he had not consumed any alcohol or illegal drugs. Moreover, he did not take any prescription drugs and neither was he suffering from a psychiatric or somatic disorder. Therefore, it seems unlikely that he suffered from a deranged hippocampus during the fatal incident. Randy said that he had complete amnesia for the stabbing. Thus, he did not report any islands of memory. His score on the SIMS was 32, indicating a strong indication of a tendency to feign psychiatric symptoms and cognitive impairments. When the police started their investigation, they had no clear picture regarding the manner in which the offense was committed. Therefore, they asked the Dutch Forensic Institute (NFI) to reconstruct the crime by analyzing forensic evidence. Using blood spatter patterns, the wounds on the victim's body, and other physical evidence, the NFI was able to almost completely reconstruct the offense. This information was not provided to Randy or his attorney. Based on the crime reconstruction, an SVT consisting of 25 two choice questions was created. Each question was followed by a correct and an incorrect answer. These 25 questions were given to a panel of 10 forensic psychologists, who were asked to give the most plausible answer to each question. This procedure showed that five of the questions did not contain two equally plausible answering options. Thus, the final SVT consisted of 20 questions. One of these questions was: “The victim was stabbed: (a) one time in her chest, two times in her neck, or (b) two times in her chest and one time in her neck.” Randy gave wrong answers to 14 of the 20 items. According to binomial statistics, the probability that his response pattern was based on random guessing was < 6 percent, indicating that there is a < 6 percent chance that his amnesia was genuine. Taken together, there was converging evidence that Randy had feigned his amnesia for the stabbing. The court also found his amnesia claim not credible. He was sentenced to 12 years imprisonment.

## Discussion

There are multiple strategies for forensic psychologists and psychiatrists to examine the veracity of crime-related amnesia claims. When asked to evaluate such claims, it would be best to use a multi-method approach (49). Especially in cases where offenders might have suffered from a deranged hippocampus at the time of the crime, forensic psychologists, and psychiatrists are advised to exercise restraint in labeling memory loss for a crime as non-credible. Only when there is converging evidence for feigning, crime-related amnesia may be deemed not authentic (25).

In order to determine whether or not the offender suffered from a deranged hippocampus at the time of the offense, a forensic psychologist or psychiatrist should have solid knowledge of neuropsychology and psychopharmacology. Although clinical features of the amnesia may yield important information about the authenticity of the memory loss reported by the offender, they cannot always be used. Because offenders may have intimate knowledge of memory loss, those who report bona-fide symptoms of amnesia may still be feigning their amnesia. Tests may shed important light on the veracity of memory loss for a crime. However, when an offender does not have a reason to feign memory problems during the forensic evaluation (e.g., an individual who claims that he or she cannot remember crime-related events because of alcohol or drug intoxication), a normal score on the SIMS, the TOMM or a related instrument does not say much about the veracity of the amnesia claim. In such cases, it would be informative to develop and administer an SVT to determine the authenticity of the memory loss reported by the offender.

## Author contributions

The author confirms being the sole contributor of this work and has approved it for publication.

### Conflict of interest statement

The author declares that the research was conducted in the absence of any commercial or financial relationships that could be construed as a potential conflict of interest.
